# Author Correction: An intein-split transactivator for intersectional neural imaging and optogenetic manipulation

**DOI:** 10.1038/s41467-024-55713-w

**Published:** 2025-01-07

**Authors:** Hao-Shan Chen, Xiao-Long Zhang, Rong-Rong Yang, Guang-Ling Wang, Xin-Yue Zhu, Yuan-Fang Xu, Dan-Yang Wang, Na Zhang, Shou Qiu, Li-Jie Zhan, Zhi-Ming Shen, Xiao-Hong Xu, Gang Long, Chun Xu

**Affiliations:** 1 Institute of Neuroscience, State Key Laboratory of Neuroscience, Center for Excellence in Brain Science and Intelligence Technology, Chinese Academy of Sciences, Shanghai, 200031 China; 2 University of the Chinese Academy of Sciences, Beijing, 100049 China; 3 Institut Pasteur of Shanghai, Chinese Academy of Sciences, Shanghai, 200031 China; 4 Beijing Advanced Innovation Center for Structural Biology, Tsinghua University, Beijing, 100084 China; 5 School of Life Science and Technology, ShanghaiTech University, Shanghai, 201210 China; 6 Shanghai Center for Brain Science and Brain-Inspired Intelligence Technology, Shanghai, 201210 China

**Keywords:** Synthetic biology, Neural circuits

Correction to: *Nature Communications* 10.1038/s41467-022-31255-x, published online 23 June 2022

In the Acknowledgements section of this article, the grant number relating to the National Key R&D Program of China given for Chun Xu was inadvertently omitted and should have been 2020YFE0205900. The original article has been updated.

