# Supplementary material for: An intein-split transactivator for intersectional neural imaging and optogenetic manipulation
Source: Nat Commun. 2022 Jun 23;13:3605. doi: 10.1038/s41467-022-31255-x (PMC9226064; doi:10.1038/s41467-022-31255-x)
Supplement: Supplementary file 1 — Supplementary Information [file 41467_2022_31255_MOESM1_ESM.pdf]

Supplementary Information for

An intein-split transactivator for intersectional neural imaging and optogenetic manipulation

Hao-Shan Chen, Xiao-Long Zhang, Rong-Rong Yang, Guang-Ling Wang, Xin-Yue Zhu, Yuan-Fang Xu, Dan-Yang Wang, Na Zhang, Shou Qiu, Li-Jie Zhan, Zhi-Ming Shen, Xiao-Hong Xu, Gang Long* and Chun Xu*

*Correspondence to: glong@ips.ac.cn; chun.xu@ion.ac.cn

This PDF file includes:

Supplementary Figure 1. The specific reconstitution of rtTA *in vitro*.

Supplementary Figure 2. The histology of IBIST-based optogenetic opsins.

Supplementary Figure 3. The position of optical fiber tips in behavioral experiments.

Supplementary Figure 4. The IBIST-based Ca^{2+} recording by GCaMP6s.

Supplementary Figure 5. The IBIST-based fluorophore labeling by two features.

Supplementary Figure 6. The IBIST-based optogenetic manipulation for cells defined by two features.

Supplementary Figure 7. Biophysical properties of vCA1 projection cells.

Supplementary Figure 8. Behavioral summary of CPP.

Supplementary Figure 9. IBIST-based optogenetic manipulation of hippocampal cells in behaving animals.

Supplementary Figure 10. Multiple-feature strategy by IBIST.

Supplementary Table 1. Electrophysiological properties of retrobeads-label cells in the vCA1.

Supplementary Table 2. Emotional stimuli-evoked Ca^{2+} responses in different types of projectors in vCA1.

Supplementary Table 3. The statistical analysis of Ca^{2+} responses in Figure 5.

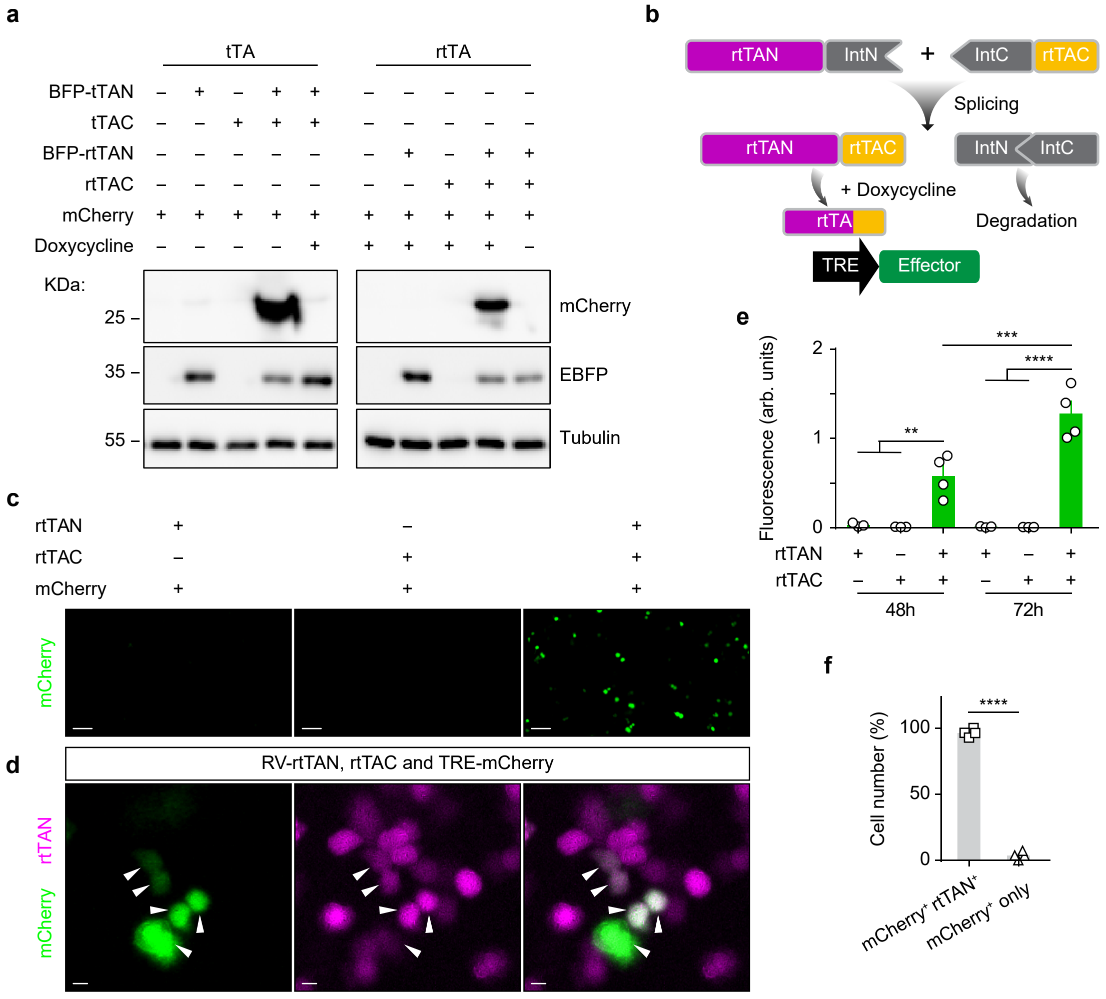

Supplementary Figure 1. The specific reconstitution of rtTA *in vitro*.
a, Western blot analysis of mCherry and BFP tagged tTA or rtTA after plasmids transfection in HEK293T cells. Uncropped blots in Source Data. **b**, Diagram depicting the reconstitution of rtTA by intein-based protein splicing. **c**, Examples of mCherry fluorescence (shown in green) in HEK293T cells 72 h after transfection with reporter (TRE-mCherry) and rtTA plasmids and infection with or without RV-rtTAN (GFP tagged). Scale bars: 100 μm. **d**, Examples showing the fluorescence of TRE-mCherry (shown in green) and RV-rtTAN (shown in magenta) in HEK293T cells with all plasmids transfected. Arrows: co-labeled cells. Scale bars: 10 μm. **e**, Summary of red fluorescence intensity of HEK293T cells at 48 h and 72 h post transfection in **c** (RV-rtTAN only, n = 3 FOV; rtTAC only, n = 3 FOV; rtTAC+RV-rtTAN, n = 4 FOV). One-way ANOVA revealed a significant difference between groups ($F_{(5,14)}$ = 34.39, ****P = 2.2×10^{-7}). Tukey's multiple comparisons test revealed that fluorescence intensity in rtTAC+RV-rtTAN group at 72 h is significantly higher than that at 48 h post transfection (***P = 0.0006), and both are significantly higher than other groups at the same time post transfection (**P < 0.01 for 48 h and ****P < 0.0001 for 72 h). **f**, Percentage summary of cells labeled by TRE-mCherry and RV-rtTAN in d (96.4 ± 1.5% co-labeled vs. 3.6 ± 1.5% reporter only; paired t-test, ****P = 6.8×10^{-5}, n = 4 FOV). Data summary: mean ± SEM. Statistical tests: two-sided. Source data are provided as a Source Data file.

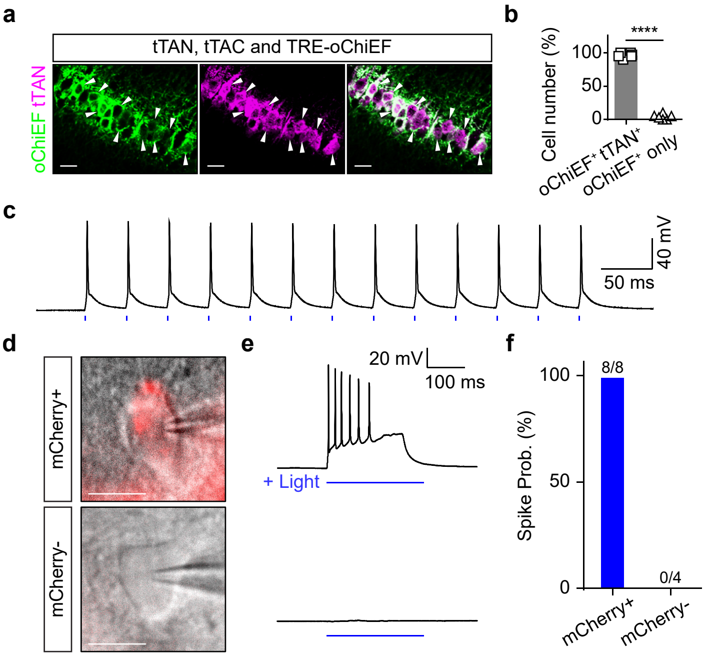

Supplementary Figure 2. The histology of IBIST-based optogenetic opsins.
a, Confocal images showing fluorescent labeling by AAV-CaMKIIα-tTAN-BFP (shown in magenta) and AAV-TRE-oChiEF-mCherry (shown in green) in hippocampal sections from animal injected with all three AAVs. Scale bars: 20 μm. **b**, Percentage summary of cells labeled by AAV-TRE-oChiEF and AAV-CaMKIIα-tTAN-BFP (96.2 ± 1.6% co-labeled vs. 3.8 ± 1.6% reporter only; paired *t*-test, ****P = 8.12×10^{-7}, n = 6 FOV, N = 2 animals). **c**, Example showing light-evoked spikes in hippocampal cells infected with AAV-TRE-oChiEF and AAVs (CaMKIIα promoter) of tTAN and tTAC. Blue ticks indicate blue LED light pulses (3 mW, 2 ms) at 25 Hz. Similar recordings were replicated in 3 cells. **d**, Examples showing mCherry fluorescence of patched cells in brain slices from animals with same injection as in **a**. Scale bars: 10 μm. **e**, Examples showing whole-cell current-clamp recording from mCherry positive and negative cells in brain slices with blue LED light stimulation (blue line). **f**, Summary of light-evoked spike probabilities (prob.) in patched cells as in **e** from 4 animals. Data summary: mean ± SEM. Statistical tests: two-sided. Source data are provided as a Source Data file.

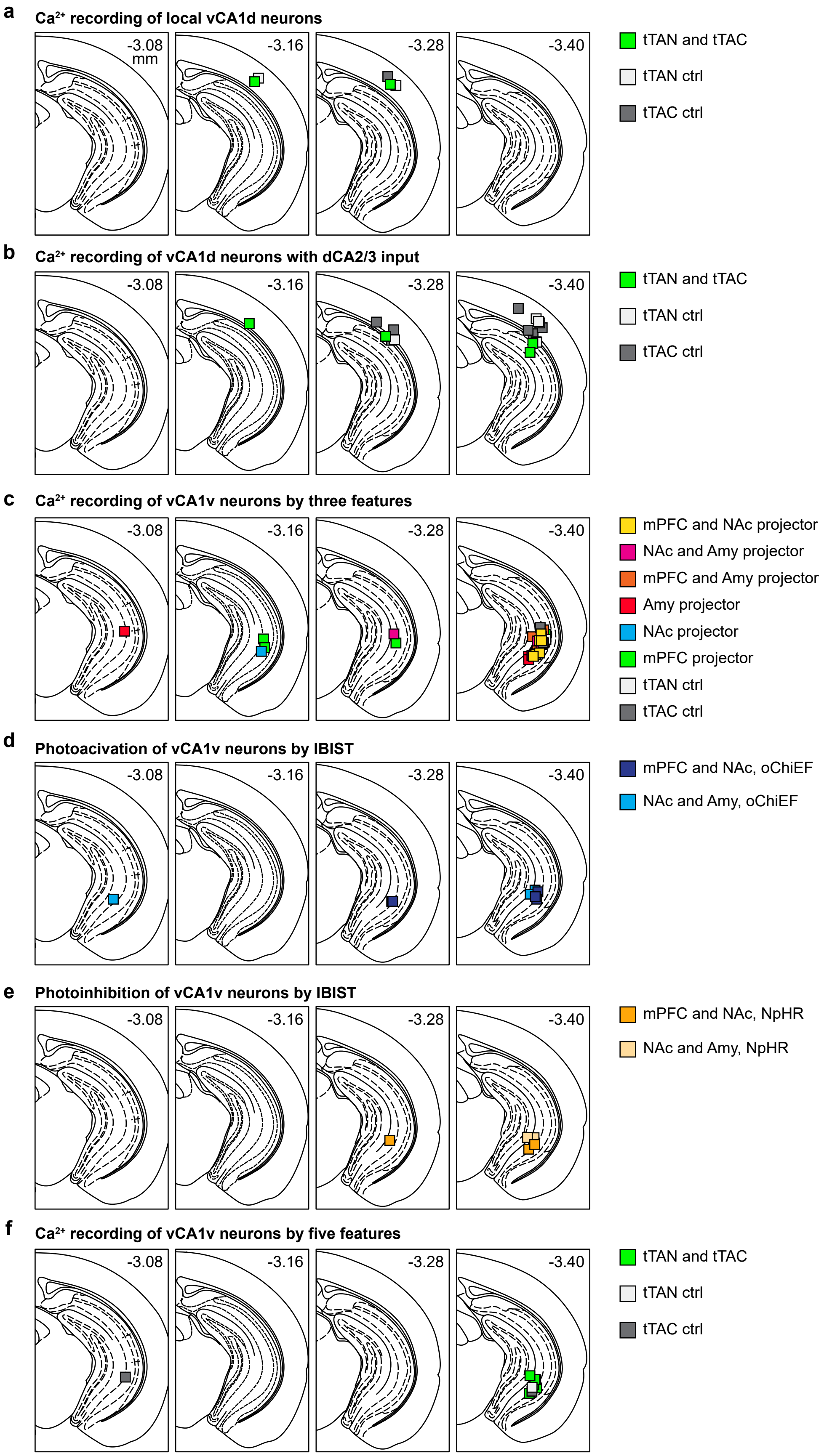

Supplementary Figure 3. The position of optical fiber tips in behavioral experiments.
a - f, Positions of implanted optical fiber tips in animals with Ca^{2+} recording (tTAN ctrl, light gray; tTAC ctrl, dark gray; tTAN and tTAC, green; mPFC projector, green; NAc projector, blue; Amy projector, red; mPFC and Amy projector, orange; NAc and Amy projector, magenta; mPFC and NAc projector, yellow) and optogenetic manipulations (mPFC and NAc projector, oChiEF, dark blue; NAc and Amy projector, oChiEF, light blue; mPFC and NAc projector, NpHR, dark yellow; NAc and Amy projector, NpHR, light yellow).

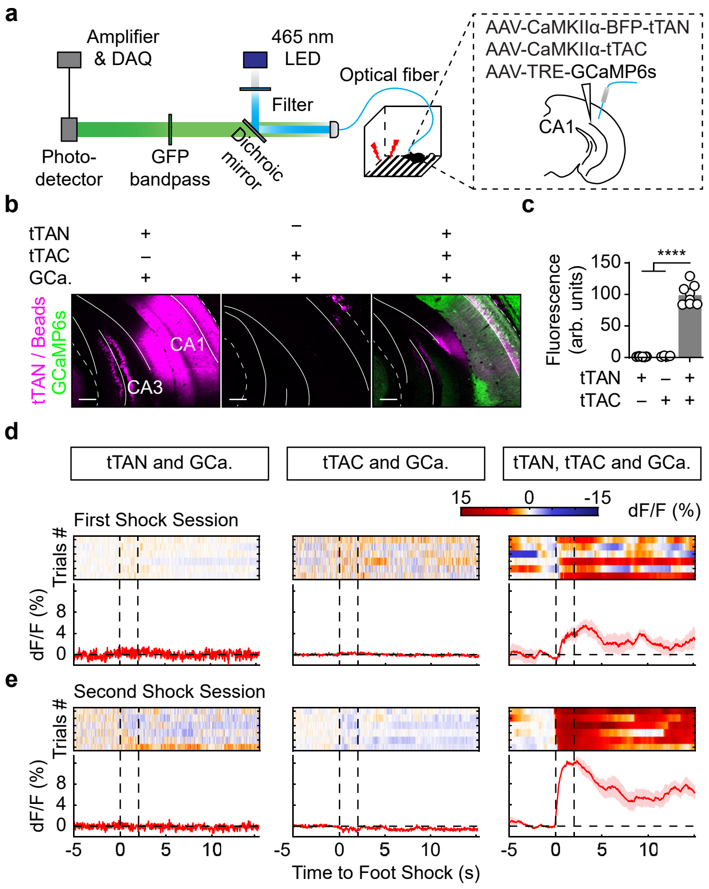

Supplementary Figure 4. The IBIST-based Ca^{2+} recording by GCaMP6s.

a, Scheme depicting the photometric recording in vCA1 injected with AAVs of TRE-GCaMP6s, CaMKIIα-tTAN and CaMKIIα-tTAC. **b**, Examples showing fluorescence of hippocampal slice from animal injected with part or all AAVs of tTAN (tagged with BFP, shown in magenta), tTAC and TRE-GCaMP6s (GCa., shown in green) and co-injected with blue beads (shown in magenta). Scale bars: 200 μm. **c**, Summary of the fluorescent intensity in **b**. One-way ANOVA revealed significant fluorescence differences between groups ($F_{(2, 17)}$ = 208.2, ****P = 1.11×10^{-12}) and Turkey's multiple comparisons revealed that the fluorescence is significantly higher in tTAN+tTAC group than in others (tTAN vs. tTAN+tTAC, ****P = 2.67×10^{-12}; tTAC vs. tTAN+tTAC, ****P = 7.85×10^{-11}). tTAN, N = 2 animals; tTAC, N = 1 animals; tTAN+tTAC, N = 2 animals. **d and e**, Examples of heatmaps and averaged traces of Ca^{2+} signals upon foot shocks (between vertical dash lines, 6 trials) in the first (**d**) and second (**e**) shock session recorded from animals injected in **b**. Similar histological and photometric recording results were obtained from 1 – 2 replicates in each group (tTAC, N= 1 animal; tTAN, N= 2 animals; tTAC+tTAN, N= 2 animals). Data summary: mean ± SEM. Statistical tests: two-sided. Source data are provided as a Source Data file.

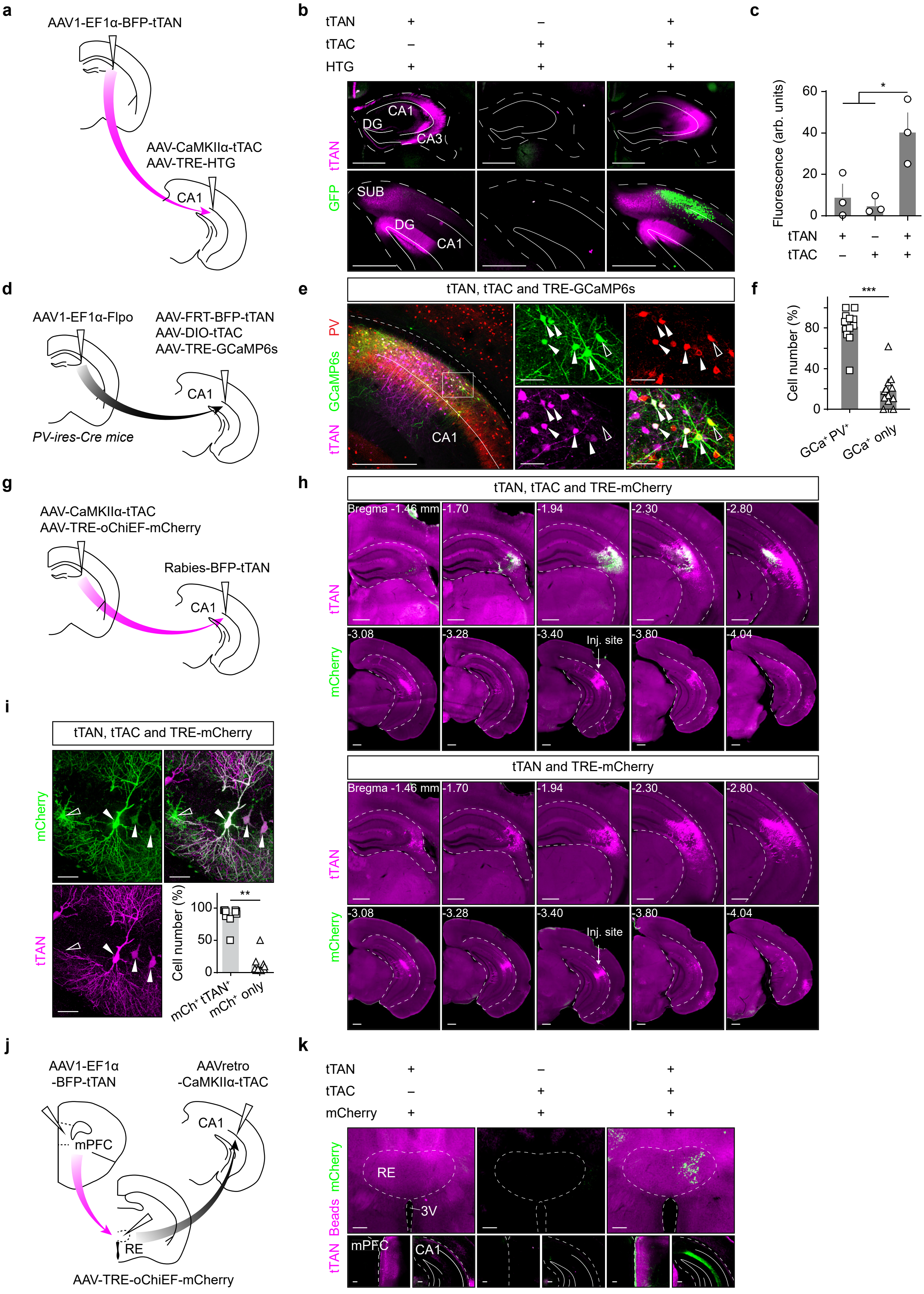

Supplementary Figure 5. The IBIST-based fluorophore labeling by two features.

Supplementary Figure 5. The IBIST-based fluorophore labeling by two features.

a, Scheme illustrating AAV injections to define two-feature cells by anterograde tracing and pyramidal-cell-specific fluorophore labeling. **b** and **c**, Examples of fluorescent labeling (**b**) and Summary of fluorescent intensity of reporter GFP (**c**). One-way ANOVA revealed significant fluorescence differences between groups ($F_{(2, 6)}$ = 9.187, *P = 0.015) and Turkey's multiple comparisons revealed that the fluorescence is significantly higher in tTAN+tTAC group than in others (tTAN vs. tTAN+tTAC, *P = 0.031; tTAC vs. tTAN+tTAC, *P = 0.018). N = 3 animals/group. **d**, Scheme illustrating AAV injections to combine anterograde transsynaptic tracing with PV+ interneuron-specific fluorophore labeling. **e**, Examples showing fluorescence in vCA1 from PV-ires-Cre animal injected with all the AAVs and enlarged pictures showing co-labeling by PV+ cells (shown in red) and AAV-TRE-GCaMP6s (shown in green). Filled arrow, double labeled. **f**, Percentage summary of cells labeled by PV immunostaining and AAV-TRE-GCaMP6s (81.6 ± 4.8% co-labeled vs. 18.4 ± 4.8% reporter only; Wilcoxon matched-pairs signed rank test, ***P = 0.001, n = 12 FOV, N = 3 animals). **g**, Scheme illustrating AAV injections to combine retrograde transsynaptic rabies tracing with pyramidal-cell-specific fluorophore labeling. **h**, Examples showing dorsal and ventral hippocampus cells labeled by rabies-BFP-tTAN (shown in magenta) and AAV-TRE-oChiEF-mCherry (shown in green) with or without AAV-CaMKIIα-tTAC injection. The arrows indicate injection site. **i**, Examples and summary showing fluorescent labeling from animals in **h** (top). Open arrow, reporter only. Filled arrow, double labeled. 88.6 ± 4.5% of mCherry+ cells were co-labeled by rabies-BFP-tTAN and 11.4 ± 4.5% were not co-labeled (Wilcoxon matched-pairs signed rank test, **P = 0.0039, n = 10 FOV, N = 4 animals). **j**, Scheme illustrating AAV injections to label cells in the nucleus of reunien (RE) connecting mPFC and vCA1. **k**, Examples showing mCherry and tTAN fluorescence in RE, mPFC and vCA1 (Replications: tTAN, N = 2 animals; tTAC, N = 2 animals; tTAN+tTAC, N = 4 animals). Scale bars: 500 μm (**e** & **h**), 50 μm (inset of **e** & **i**), 200 μm (**b** & **k**). Data summary: mean ± SEM. Statistical tests: two-sided. Source data are provided as a Source Data file.

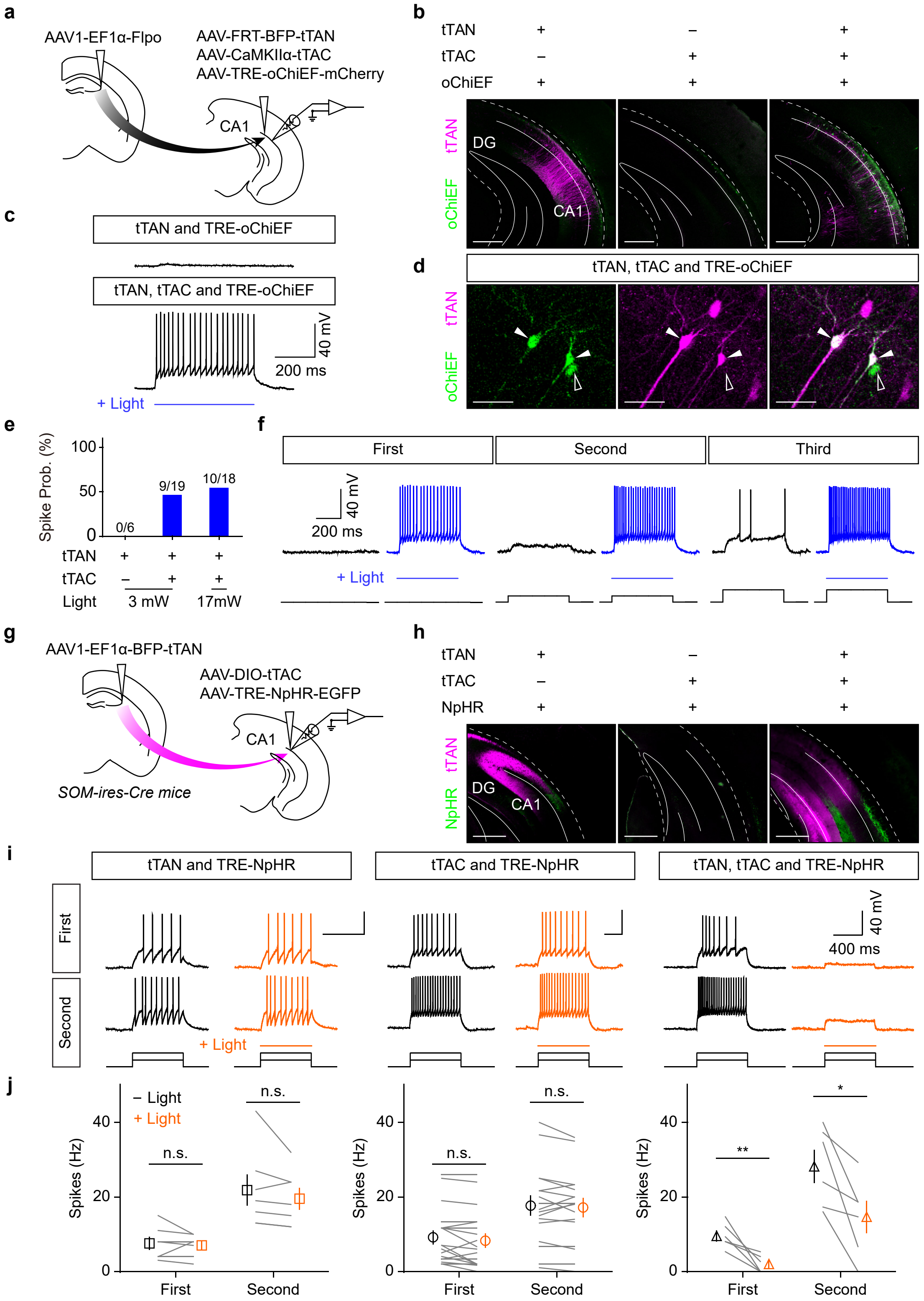

Supplementary Figure 6. The IBIST-based optogenetic manipulation for cells defined by two features.
a, Scheme illustrating AAV injections to label two-feature cells by anterograde tracing and pyramidal-cell-specific expression of ChR2. **b**, Fluorescence examples in ventral hippocampus injected with AAVs of tTAN (magenta), tTAC or TRE-oChiEF-mCherry (green). **c**, Examples showing whole-cell current-clamp recording in brain slices with blue LED light stimulation (line). **d**, Examples showing fluorescent labeling by AAV-FRT-BFP-tTAN (magenta) and AAV-TRE-oChiEF-mCherry (green) in hippocampal slices. Open arrow, reporter only. Filled arrow, double labeled. **e**, Summary of spike probabilities (prob.) in hippocampal cells evoked by whole-field blue LED light: tTAN, N = 2 animals; tTAN+tTAC, 47% at 3 mW, 56% at 17 mW, N = 4 animals. **f**, Examples showing whole-cell current-clamp recording with or without blue light (0, 40 and 80 pA steps). **g**, Scheme illustrating AAV injections to combine anterograde tracing with SOM-interneuron-specific expression of NpHR in SOM-ires-Cre mice. **h**, Fluorescence examples of hippocampus infected by AAVs. **i**, Examples showing whole-cell current-clamp recording from hippocampal cells upon current injections (40 pA step) with or without light (first and second sweeps with prominent spikes). **j**, Summary of current-evoked spike frequency in the absence (black) or presence (yellow) of 589 nm light (18 mW). Paired *t*-test: AAV-tTAN (first sweep, 7.6 ± 1.6 Hz vs. 7.0 ± 1.1 Hz with light; P = 0.59; second sweep, 21.9 ± 4.0 Hz vs. 19.6 ± 2.8 Hz with light; P = 0.19, n = 6 cells, N = 2 animals), AAV-tTAC (first sweep, 9.2 ± 1.7 Hz vs. 8.3 ± 1.8 Hz with light; P = 0.09; second sweep, 17.7 ± 2.6 Hz vs. 17.2 ± 2.4 Hz with light; P = 0.37, n = 18 cells, N = 4 animals) or both (first sweep, 9.6 ± 1.4 Hz vs. 2.0 ± 1.0 Hz with light; **P = 0.0087; second sweep, 28.2 ± 4.3 Hz vs. 14.7 ± 4.2 Hz with light; *P = 0.02, n = 6 cells, N = 2 animals). Scale bars: 500 µm (**b**, **h**), 50 µm (**d**). Data summary: mean ± SEM. Statistical tests: two-sided. Source data are provided as a Source Data file.

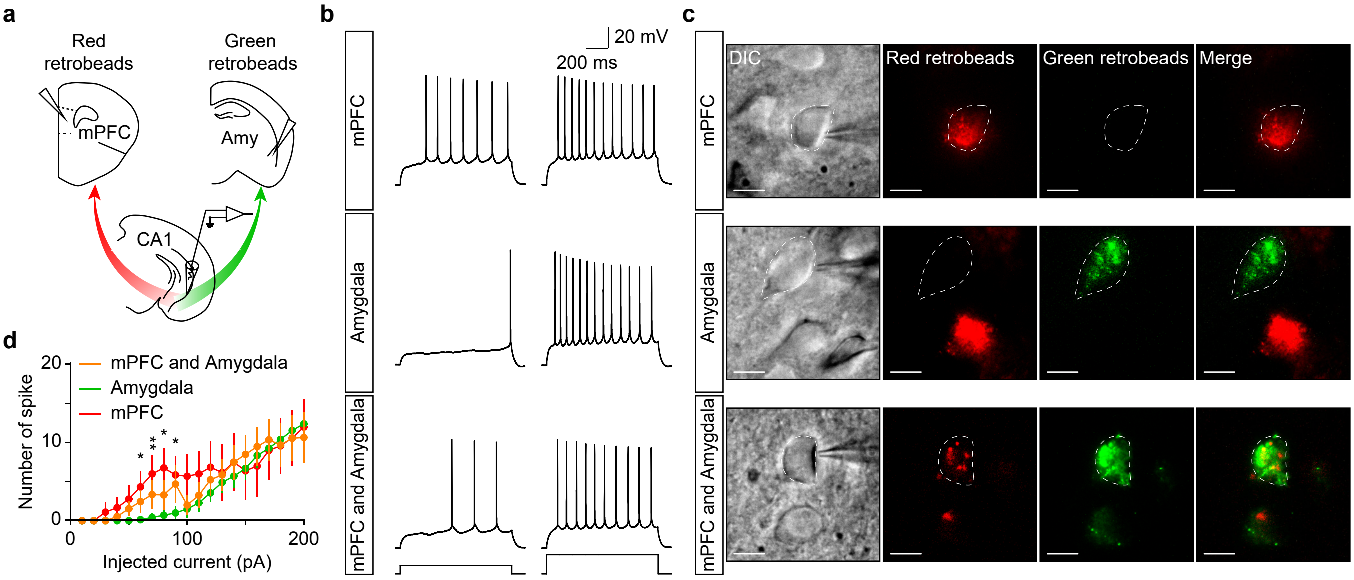

Supplementary Figure 7. Biophysical properties of vCA1 projection cells.
a, Scheme illustrating injections to label vCA1 cells projecting to mPFC, amygdala, or both. **b**, Examples showing whole-cell current-clamp recording from vCA1 projection cells upon current injections (left, 80 pA; right, 200 pA). **c**, Examples showing fluorescence of red and green retrobeads in vCA1 cells in acute brain slices and differential interference contrast (DIC) images. Scale bars: 10 µm. **d**, Summary (mean ± SEM) of spike numbers evoked by current injections in vCA1 projection cells (amygdala projectors: n = 14 cells; mPFC projectors: n = 9 cells; amygdala-mPFC projectors, n = 11 cells; N = 9 animals). Statistics tests: two-sided unpaired *t*-test, *P < 0.05, **P < 0.01, details are in Supplementary table 1. Source data are provided as a Source Data file..

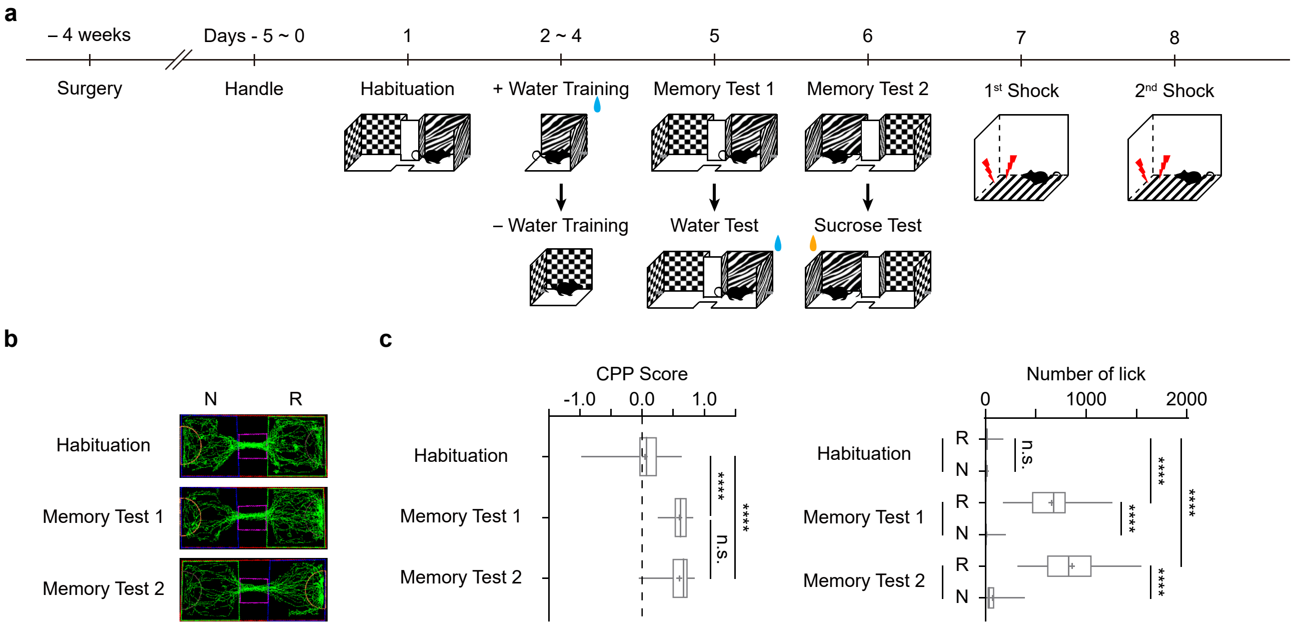

Supplementary Figure 8. Behavioral summary of CPP.

a, Scheme illustrating behavioral training protocols for CPP and foot shock sessions. **b**, Examples showing trajectories during test sessions of water CPP. N, non-rewarding context. R, rewarding context. **c**, Summary of CPP score and lick number during test sessions. N = 40 animals. One-way ANOVA revealed a significant difference in CPP score ($F_{(1.579,\ 61.59)}$ = 84.29, ****P = 1.8×10^{-16}) and lick number ($F_{(2.022,\ 78.84)}$ = 212.9, ****P = 1.2×10^{-32}) after training. Tukey's multiple comparisons test, ****P < 0.0001. Statistical tests: two-sided. Box plots: whiskers (min/max), middle line (median), plus (mean), box (25/75 percentile). Source data are provided as a Source Data file.

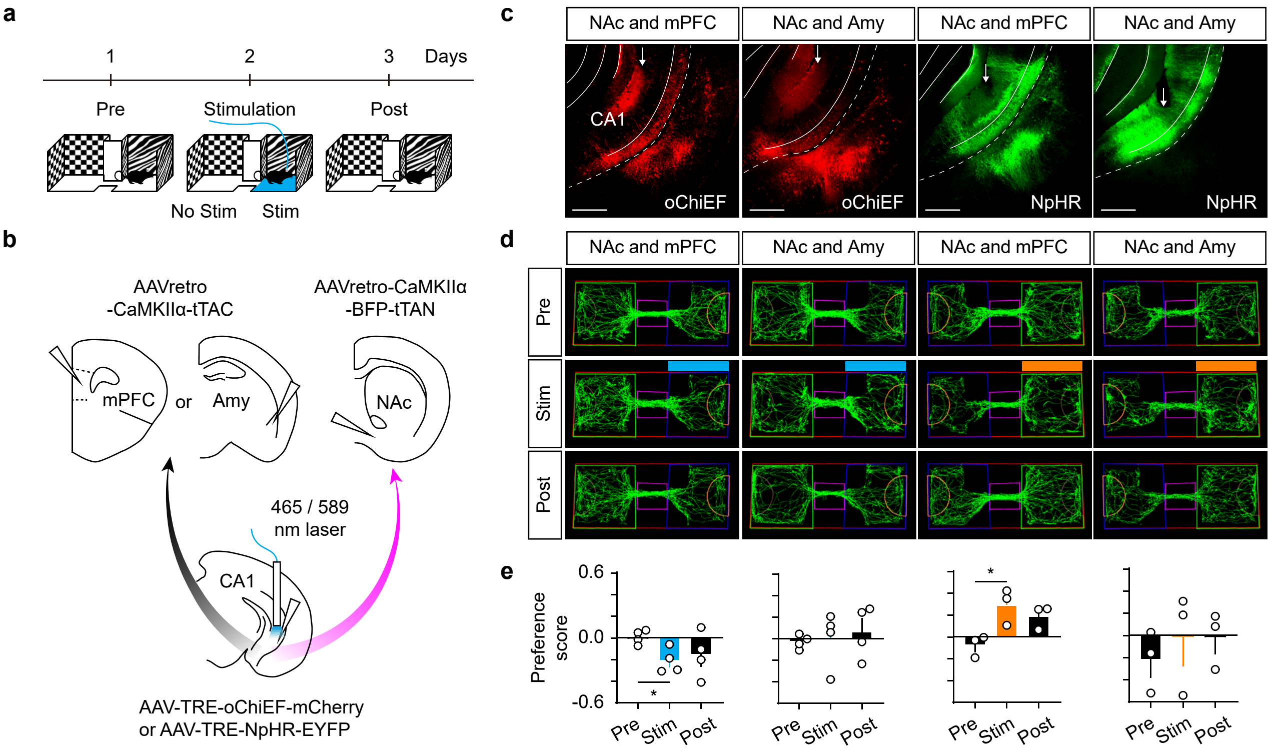

Supplementary Figure 9. IBIST-based optogenetic manipulation of hippocampal cells in behaving animals.
a, Scheme illustrating behavioral protocol. **b**, Scheme illustrating AAV injections to define subpopulations of vCA1 cells. **c**, Examples of fluorescence images in the vCA1. The arrows indicate optical fiber tracks. Scale bars: 500 µm. **d**, Examples showing animal trajectories during real-time place preference test. Blue and yellow bars indicate blue and yellow laser stimulation in specific contexts, respectively. **e**, Summary of preference score in day 1 (pre), day 2 (optogenetic stimulation) and day 3 (post). Activation: NAc and mPFC projector: Pre, 0.01 ± 0.03; Stim, -0.21 ± 0.06; Post, -0.16 ± 0.11; Paired *t*-test, Pre vs. Stim, *P = 0.043; Pre vs. Post, P = 0.21. N = 4 animals; NAc and Amy projector: Pre, -0.03 ± 0.03; Stim, 0.001 ± 0.13; Post, 0.07 ± 0.12; Paired *t*-test, Pre vs. Stim, P = 0.79; Pre vs. Post, P = 0.38. N = 4 animals. Inhibition: NAc and mPFC projector: Pre, -0.08 ± 0.06; Stim, 0.29 ± 0.1; Post, 0.19 ± 0.06; Paired *t*-test, Pre vs. Stim, *P = 0.011; Pre vs. Post, P = 0.12. N = 3 animals; NAc and Amy projector: Pre, -0.22 ± 0.16, Stim, -0.02 ± 0.27, Post, -0.02 ± 0.15; Paired *t*-test, Pre vs. Stim, P = 0.22; Pre vs. Post, P = 0.13. N = 3 animals). Data summary: mean ± SEM. Statistical tests: two-sided. Source data are provided as a Source Data file.

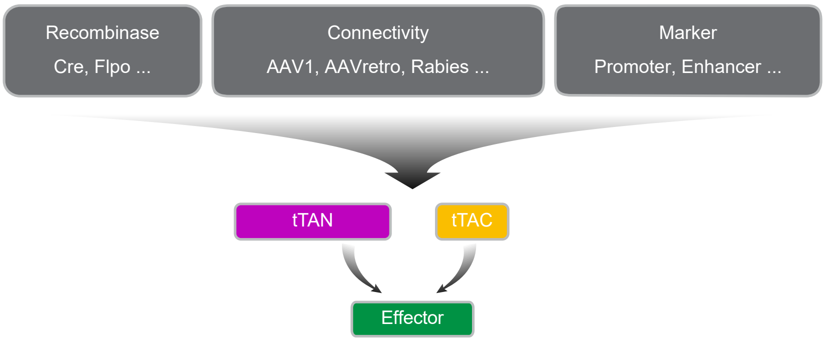

Supplementary Figure 10. Multiple-feature strategy by IBIST.
Scheme illustrating strategies for IBIST-based labeling cells via multiple features. There are two layers of controllers: one consists of intein-split tTA fragments; the other are recombinase, viral tracer and molecular markers. Similar design is also applicable for intein-split rtTA fragments. Both tTA and rtTA offer an additional feature to be controlled by doxycycline.

	Mean ± S.E.M.			P value		
Parameters	mPFC	Amy	mPFC and Amy	mPFC vs. Amy	mPFC vs. mPFC and Amy	Amy vs. mPFC and Amy
Vm (mV)	-65.3 ± 1.4	-63.9 ± 0.9	-65.4 ± 1.0	0.35	0.96	0.26
Rm (mΩ)	153.1 ± 31.6	95.0 ± 5.4	104.6 ± 11.8	0.036	0.14	0.43
Cm (pF)	142.8 ± 12.4	189.6 ± 20.0	176.6 ± 17.5	0.098	0.15	0.64
Tau (ms)	20.3 ± 3.2	17.6 ± 1.6	18.4 ± 2.6	0.42	0.65	0.79
Vth (mV)	-36.3 ± 1.5	-36.6 ± 0.8	-34.3 ± 1.2	0.83	0.31	0.11
Rheobase (pA)	87.8 ± 16.9	115.0 ± 8.1	104.6 ± 17.4	0.12	0.5	0.56
Spike peak (mV)	41.4 ± 1.9	47.6 ± 1.0	43.8 ± 1.1	0.0045	0.27	0.017
Spike amplitude (mV)	77.7 ± 2.4	84.3 ± 1.3	78.1 ± 1.9	0.017	0.91	0.01
Spike half-height duration (ms)	1.4 ± 0.1	1.3 ± 0.04	1.4 ± 0.1	0.3	0.94	0.098
Spike rise (ms)	0.4 ± 0.04	0.4 ± 0.02	0.7 ± 0.3	0.21	0.41	0.24
Spike decay (ms)	2.9 ± 0.8	1.8 ± 0.3	1.7 ± 0.2	0.2	0.14	0.79

Supplementary Table 1. Electrophysiological properties of retrobeads-label cells in the vCA1.

Data are presented as mean ± SEM. The significance between two groups were determined by two-sided unpaired *t*-test. mPFC, medial prefrontal cortex. Amy, amygdala.

Emotional stimuli	Water	Sucrose	Water omission	1st shock session	2nd shock session
NAc (N = 5)	0.23 ± 0.05%	0.34 ± 0.11%	-0.32 ± 0.06%	5.0 ± 1.1%	3.9 ± 0.7%
Amy and NAc (N = 6)	0.12 ± 0.07%	0.52 ± 0.16%	-0.09 ± 0.09%	4.5 ± 1.0%	3.6 ± 1.3%
Amy (N = 6)	0.01 ± 0.08%	0.28 ± 0.1%	-0.28 ± 0.06%	6.1 ± 0.8%	4.4 ± 0.6%
mPFC and Amy (N = 5)	0.06 ± 0.04%	0.1 ± 0.03%	-0.06 ± 0.05%	1.8 ± 0.3%	1.7 ± 0.9%
mPFC (N = 6)	0.04 ± 0.03%	0.15 ± 0.09%	-0.19 ± 0.07%	0.9 ± 0.6%	0.8 ± 0.6%
NAc and mPFC (N = 6)	-0.01 ± 0.07%	0.05 ± 0.07%	-0.12 ± 0.06%	2.7 ± 0.6%	1.8 ± 0.6%
tTAC (Ctrl, N = 3)	-0.07 ± 0.07%	0.00 ± 0.00%	0.19 ± 0.19%	-0.1 ± 0.1%	0.0 ± 0.0%
tTAN (Ctrl, N = 3)	0.00 ± 0.00%	0.04 ± 0.04%	0.00 ± 0.00%	0.1 ± 0.1%	-0.5 ± 0.5%

Supplementary Table 2. Emotional stimuli-evoked Ca^{2+} responses in different types of projectors in vCA1. Related to Figure 5. Data are presented as mean ± SEM. N indicates animal number. NAc, nucleus accumbens. Amy, amygdala. mPFC, medial prefrontal cortex.

Emotional stimuli	Water	Sucrose	Water omission	1st shock session	2nd shock session
**One sample *t*-test (H0: μ = 0)**					
NAc	0.0091	0.035	0.0069	0.0095	0.005
Amy and NAc	0.13	0.021	0.36	0.0079	0.041
Amy	0.92	0.042	0.0079	0.0006	0.001
mPFC and Amy	0.20	0.019	0.31	0.0057	0.16
mPFC	0.21	0.16	0.042	0.18	0.25
NAc and mPFC	0.89	0.52	0.12	0.0053	0.027
tTAC (ctrl)	0.42	NA	0.42	0.42	NA
tTAN (ctrl)	NA	0.42	NA	0.42	0.42
**Unpaired *t*-test**					
NAc vs. Amy/NAc	0.21	0.39	0.082	0.74	0.84
NAc vs. Amy	0.047	0.69	0.65	0.43	0.65
NAc vs. mPFC/Amy	0.024	0.062	0.012	0.023	0.09
NAc vs. mPFC	0.0055	0.20	0.22	0.0057	0.0074
NAc vs. NAc/mPFC	0.022	0.043	0.055	0.074	0.039
NAc vs. tTAC (ctrl)	0.011	0.056	0.019	0.012	0.0058
NAc vs. tTAN (ctrl)	0.012	0.083	0.0086	0.014	0.0046
Amy/NAc vs. Amy	0.31	0.22	0.13	0.25	0.61
Amy/NAc vs. mPFC/Amy	0.48	0.039	0.78	0.055	0.28
Amy/NAc vs. mPFC	0.29	0.066	0.41	0.012	0.08
Amy/NAc vs. NAc/mPFC	0.20	0.02	0.81	0.16	0.23
Amy/NAc vs. tTAC (ctrl)	0.12	0.058	0.17	0.021	0.10
Amy/NAc vs. tTAN (ctrl)	0.26	0.074	0.52	0.025	0.076
Amy vs. mPFC/Amy	0.60	0.16	0.032	0.0013	0.036
Amy vs. mPFC	0.72	0.36	0.40	0.0003	0.0021
Amy vs. NAc/mPFC	0.86	0.093	0.12	0.0057	0.012
Amy vs. tTAC (ctrl)	0.56	0.11	0.02	0.0011	0.0022
Amy vs. tTAN (ctrl)	0.94	0.16	0.022	0.0013	0.0017
mPFC/Amy vs. mPFC	0.67	0.63	0.18	0.19	0.44
mPFC/Amy vs. NAc/mPFC	0.42	0.55	0.50	0.26	0.91
mPFC/Amy vs. tTAC (ctrl)	0.13	0.029	0.16	0.0059	0.24
mPFC/Amy vs. tTAN (ctrl)	0.29	0.20	0.42	0.01	0.16
mPFC vs. NAc/mPFC	0.52	0.40	0.47	0.046	0.26
mPFC vs. tTAC (ctrl)	0.12	0.29	0.05	0.29	0.40
mPFC vs. tTAN (ctrl)	0.36	0.42	0.11	0.39	0.22
NAc/mPFC vs. tTAC (ctrl)	0.62	0.65	0.088	0.013	0.071
NAc/mPFC vs. tTAN (ctrl)	0.92	0.91	0.25	0.018	0.04

Supplementary Table 3. The statistical analysis of Ca^{2+} responses in Figure 5.

Two-sided one sample *t*-test and unpaired *t*-test were performed for statistical comparison. NAc, nucleus accumbens. Amy, amygdala. mPFC, medial prefrontal cortex.
