# Supplementary material for: An intein-split transactivator for intersectional neural imaging and optogenetic manipulation
Source: Nat Commun. 2022 Jun 23;13:3605. doi: 10.1038/s41467-022-31255-x (PMC9226064; doi:10.1038/s41467-022-31255-x)
Supplement: Supplementary file 3 — Reporting Summary [file 41467_2022_31255_MOESM3_ESM.pdf]

nature research

Corresponding author(s): Dr. Gang Long
Dr. Chun Xu

Last updated by author(s): May 1, 2022

Reporting Summary

Nature Research wishes to improve the reproducibility of the work that we publish. This form provides structure for consistency and transparency in reporting. For further information on Nature Research policies, see our Editorial Policies and the Editorial Policy Checklist.

Statistics

For all statistical analyses, confirm that the following items are present in the figure legend, table legend, main text, or Methods section.

n/a	Confirmed	
☐	☒	The exact sample size (n) for each experimental group/condition, given as a discrete number and unit of measurement
☐	☒	A statement on whether measurements were taken from distinct samples or whether the same sample was measured repeatedly
☐	☒	The statistical test(s) used AND whether they are one- or two-sided<br>*Only common tests should be described solely by name; describe more complex techniques in the Methods section.*
☒	☐	A description of all covariates tested
☐	☒	A description of any assumptions or corrections, such as tests of normality and adjustment for multiple comparisons
☐	☒	A full description of the statistical parameters including central tendency (e.g. means) or other basic estimates (e.g. regression coefficient) AND variation (e.g. standard deviation) or associated estimates of uncertainty (e.g. confidence intervals)
☐	☒	For null hypothesis testing, the test statistic (e.g. F, t, r) with confidence intervals, effect sizes, degrees of freedom and P value noted<br>*Give P values as exact values whenever suitable.*
☒	☐	For Bayesian analysis, information on the choice of priors and Markov chain Monte Carlo settings
☒	☐	For hierarchical and complex designs, identification of the appropriate level for tests and full reporting of outcomes
☒	☐	Estimates of effect sizes (e.g. Cohen's d, Pearson's r), indicating how they were calculated

Our web collection on statistics for biologists contains articles on many of the points above.

Software and code

Policy information about availability of computer code

Data collection	The microscope images were captured in the respective microscope software: Neurolucida 11 for Nikon Eclipse 80i (MBF Bioscience), BioLife for CKX53 and FV31S-SW v2.3.1 for Olympus FV3000. The photometric recording was acquired in Cinelyzer (Plexon Inc). The patch-clamp recording was acquired in pClamp10 (Molecular Device).
Data analysis	Fiji (ImageJ 1.48), Imaris (v7.4.2, Bitplane), GraphPad PRISM 8, MATLAB R2018b, Clampfit 10.7.

For manuscripts utilizing custom algorithms or software that are central to the research but not yet described in published literature, software must be made available to editors and reviewers. We strongly encourage code deposition in a community repository (e.g. GitHub). See the Nature Research guidelines for submitting code & software for further information.

Data

Policy information about availability of data

All manuscripts must include a data availability statement. This statement should provide the following information, where applicable:

- Accession codes, unique identifiers, or web links for publicly available datasets
- A list of figures that have associated raw data
- A description of any restrictions on data availability

Source data are provided with this paper and publicly available at the following repository https://github.com/XuChunLab/IBIST. The plasmids created in this study are available on Addgene (#172119 – 172127).

Field-specific reporting

Please select the one below that is the best fit for your research. If you are not sure, read the appropriate sections before making your selection.

☒ Life sciences ☐ Behavioural & social sciences ☐ Ecological, evolutionary & environmental sciences

For a reference copy of the document with all sections, see nature.com/documents/nr-reporting-summary-flat.pdf

Life sciences study design

All studies must disclose on these points even when the disclosure is negative.

Sample size	The sample size was not pre-determined. The sample size was comparable with prior studies (Fenno et al., Neuron, 2020).
Data exclusions	No data was excluded for analysis.
Replication	All attempts at replication were successful. For cell culture experiments, each group typically has 4 culture plates (one FOV analyzed per culture plate). For histology and slice recording, each group typically has >6 FOV or cells from 2 - 6 mice. For in vivo photometric experiments, each group typically has 3 mice or more.
Randomization	Allocation of samples or animals was random.
Blinding	The fluorescence in culture cells and PFA fixed brain slices were so obvious between groups that the investigators were not blinded. The investigators were blinded to group allocation for animals during photometric recording and analysis.

Reporting for specific materials, systems and methods

We require information from authors about some types of materials, experimental systems and methods used in many studies. Here, indicate whether each material, system or method listed is relevant to your study. If you are not sure if a list item applies to your research, read the appropriate section before selecting a response.

Materials & experimental systems

n/a	Involved in the study	
☐	☒	Antibodies
☐	☒	Eukaryotic cell lines
☒	☐	Palaeontology and archaeology
☐	☒	Animals and other organisms
☒	☐	Human research participants
☒	☐	Clinical data
☒	☐	Dual use research of concern

Methods

n/a	Involved in the study	
☒	☐	ChIP-seq
☒	☐	Flow cytometry
☒	☐	MRI-based neuroimaging

Antibodies

Antibodies used	The primary antibodies used in this study were goat anti-GFP (1:1000, Abcam, ab6673), rabbit anti-RFP (1:1000, MBL, PM005) and guinea pig anti-PV (1:500, SYSY, 195004). The secondary antibodies used in this study were donkey anti-goat alexa fluor 488 (1:500, Invitrogen, A11055), goat anti-rabbit alexa fluor 647 (1:500, Invitrogen, A21245) and goat anti-guinea pig alexa fluor 647 (1:500, Invitrogen, A21450).
Validation	The primary antibodies we used in this study have been validated in mouse brain slices in the following peer-reviewed papers:<br><br>1. goat anti-GFP (Abcam, ab6673)<br>Xu, C et al. (2016) Distinct Hippocampal Pathways Mediate Dissociable Roles of Context in Memory Retrieval. Cell 167, 961-972.<br>2. rabbit anti-RFP (MBL, PM005)<br>Williams, S., Beronja, S., Pasolli, H. et al. Asymmetric cell divisions promote Notch-dependent epidermal differentiation. Nature 470, 353–358 (2011).<br>3. guinea pig anti-PV (SYSY, 195004)<br>Amegandjin, C.A., Choudhury, M., Jadhav, V. et al. Sensitive period for rescuing parvalbumin interneurons connectivity and social behavior deficits caused by TSC1 loss. Nat Commun 12, 3653 (2021).

Eukaryotic cell lines

Policy information about cell lines

Cell line source(s)	293T cells were purchased from National Collection of Authenticated Cell Cultures (SCSP-502). B7GG cells were kindly provided by E.M. Callaway (Salk Institute).
Authentication	The 293T cells were authenticated by the cell bank in Shanghai (https://www.cellbank.org.cn/search-detail.php?id=519). No authentication was performed for the B7GG cells by ourselves, and this cell line was already used in our previous studies (Xu et al., Cell, 2016, 167, 961–972).
Mycoplasma contamination	Cell lines tested negative for mycoplasma contamination.
Commonly misidentified lines (See ICLAC register)	No commonly misidentified cell lines were used.

Animals and other organisms

Policy information about studies involving animals; ARRIVE guidelines recommended for reporting animal research

Laboratory animals	Wild-type C57BL/6J, SOM-ires-Cre (JAX 013044) and PV-ires-Cre (JAX 017320) mice were used. All of the experimental mice used in the study were adult male mice (over 8 weeks). One male cynomolgus monkey (Macaca fascicularis, 4.2 kg, 13 years old) was obtained from non-human primate facility of Institute of Neuroscience after it was retired from the full use in reproductive research.
Wild animals	Not used.
Field-collected samples	Not used.
Ethics oversight	All animal procedures were performed in accordance with institutional guidelines and were approved by the Institutional Animal Care and Use Committee (IACUC) of the Institute of Neuroscience (CAS Center for Excellence in Brain Science and Intelligence Technology), Chinese Academy of Sciences.

Note that full information on the approval of the study protocol must also be provided in the manuscript.
